# Naturally Occurring Stilbenoid TSG Reverses Non-Alcoholic Fatty Liver Diseases via Gut-Liver Axis

**DOI:** 10.1371/journal.pone.0140346

**Published:** 2015-10-16

**Authors:** Pei Lin, Jianmei Lu, Yanfang Wang, Wen Gu, Jie Yu, Ronghua Zhao

**Affiliations:** Department of Pharmacy, Yunnan University of Traditional Chinese Medicine, Kunming, Yunnan Province, China; University of Catanzaro Magna Graecia, ITALY

## Abstract

The gut-liver axis is largely involved in the development of non-alcoholic fatty liver disease (NAFLD). We investigated whether 2, 3, 5, 4′-tetrahydroxy-stilbene-2-*O*-*β*-D-glucoside (TSG) could reverse NAFLD induced by a high-fat diet (HFD) and whether it did so via the gut-liver axis. Results showed that TSG could reduce the accumulation of FFA and it did so by reducing the expression of L-FABP and FATP4. TSG regulated gut microbiota balanced and increased the protein expression of ZO-1 and occludin, which could improve the function of the intestinal mucosal barrier and reduce serum LPS content by about 25%. TSG reduced TL4 levels by 56% and NF-κB expression by 23% relative to the NAFLD model group. This suggests that prevention of NAFLD by TSG in HFD-fed rats is mediated by modulation of the gut microbiota and TLR4/NF-κB pathway, which may alleviate chronic low-grade inflammation by reducing the exogenous antigen load on the host.

## Introduction

The term metabolic syndrome pertains to a series of diseases such as diabetes, dyslipidemia, obesity, and fatty liver disease [1]. More than 20% of the general population in developed countries is diagnosed with fatty liver disease [2]. Furthermore, 20% of fatty liver patients can develop liver cirrhosis. The life expectancy of 50% of liver cirrhosis patients is less than 10 years after diagnosis. Therefore, fatty liver disease ranks as the second most common cancer-related death in these populations [3].

The development of non-alcoholic fatty liver disease (NAFLD) has been associated with complex factors such as host genetics, diet, and lack of exercise. In addition, gut microbiota may also be involved in the development of NAFLD [4]. A growing body of evidence shows that gut microbiota causes intestinal inflammation and chronic inflammatory disease of the liver. Overgrowth of intestinal bacterial, intestinal mucosa structure damage and increased permeability are features of non-alcoholic steatohepatitis (NASH). The gut-liver axis or microbiota-liver axis pertains to the direct connection between the portal vein and the intestine and liver, which permit the transfer of gut bacteria and their products to the liver. Fat in the daily diet, together with other factors, may cause gut microbiota dysbiosis, and alterations in intestinal permeability, thus resulting in NAFLD. Studies have also shown that gut microbiota and their products accelerate the progress of NAFLD [5]. The gut microbial products appear to activate TLR (toll-like receptor), which drives the inflammation that defines disease. Microbiota can improve the extraction of energy from food, shift the body’s overall metabolism towards increased fatty free acid (FFA) absorption from adipose tissues, and transform lipid metabolism from oxidation to *de novo* production [6].

Liver diseases, including NAFLD, are related to the activation of innate immunity. Germline-encoded pattern recognition receptors such as TLRs, play an important role in recognizing pathogen-associated molecular patterns (PAMPs). These also serve as an important link between innate and adaptive immunity in early host defense. As a major component of the innate immune system, TLRs are responsible for recognizing bacterial and viral components such as lipopolysaccharides (LPSs), bacterial DNA, and peptidoglycans [7]. Due to bacterial overgrowth and increased intestinal permeability, the liver of NAFLD patients is required to process a higher amount of TLR ligands than healthy ones [7]. A previous study using gene-modified mouse models has shown that the TLR4 and TLR9 signaling pathways promote the progression of NAFLD [8]. TLR4 is the receptor for LPS, which is a component of gram-negative bacterial cell walls. Serum LPS plays a role in the maintenance of relatively high TLR levels in NAFLD patients [9]. Myeloid differentiation factor 88 (MyD88) is a downstream adaptor protein for all TLRs, except TLR3 [10]. The TLR-MyD88-NF-κB signaling cascade is crucial to the inflammatory response in immune cells and is a key to the development of NAFLD [11].

Various treatment regimens for NAFLD have been applied clinically. However, only a few have shown satisfactory results in reversing histological abnormalities or in relieving clinical symptoms. Furthermore, many of these treatment schemes induce an increase in the levels of biochemical markers such as glutamic-pyruvic transaminase and glutamic oxalacetic transaminase. Gradually decreasing body weight through diet and engaging in a sufficient amount of exercise seem to be a requirement for the treatment of NAFLD [12]. Drug treatment remains an important part of addressing these issues, which include insulin sensitizers (metformin [13] and thiazolidinediones [14]), vitamin E [15], and statins [16].

Studies have shown that plant metabolites, such as resveratrol [17] and its analogs [18] and berberine [19] can improve the general health of mammals, and they may be suitable for the treatment of age- and NAFLD-related disorders. The naturally occurring stilbenoid TSG (2, 3, 5, 4'-tetrahydroxy-stilbene-2-*O*-*β*-D-glucoside) is mainly distributed in *Polygonum multiflorum* Thunb. It has shown very good lipid regulatory effects [20]. Other studies have also shown that it has beneficial health effects [21–23]. TSG has drawn interest because it is an analog of resveratrol. The present study systematically assessed *in vivo* Spraque-Dawley rats fed on a high-fat diet (HFD) to evaluate the activities and mechanisms of TSG in the reversal of NAFLD via the gut-liver axis.

## Materials and Methods

### Experimental design

Eight-week-old male and female Spraque-Dawley rats were obtained from Chengdu, China (Certificate of Quality No: 0016254). Experiments were specially approved by the Institutional Ethical Committee on Animal Care and Experimentations of Yunnan University of Traditional Chinese Medicine (R-062014012). All reasonable efforts were made to minimize the animals’ suffering.

Rats were randomized into seven groups of 7 animals each: (CON) normal control group; (MOD) HFD induced NAFLD model group; (TSG.L) low dose of TSG (12 mg/kg); (TSG.M) middle dose of TSG (24 mg/kg); (TSG.H) high dose of TSG (48 mg/kg); (SIM) simvastatin (1.8 mg/kg) served as a positive control; (FEN) fenofibrate (18 mg/kg) served as a positive control. TSG was purchased from Nanjing Jingzhu Bio-technology Co., Ltd., China. The purity was not less than 98%. Simvastatin (Hangzhou MSD Pharmaceutical Co., Ltd., China) and fenofibrate (Laboratories Fournier S.A., France) were used as positive controls.

All rats not in the normal group were fed an HFD until the end of the experiment (12 weeks). This food contained 1% cholesterol, 10% lard, 10% eggs, and 79% basic feed (moisture ≤ 10%; protein ≥ 20%; fat mix ≥ 4%; calcium: 1.0%–1.8%; phosphorus: 0.6–1.2; fiber ≤5%; essential amino acids ≥ 2%) (Research Diets, Suzhou, China). This HFD formulation is a classic system for high-energy intake for the induction of NAFLD and hyperlipidemia in rodents [24, 25].

### Assessment of TC, TG, and lipoprotein levels in blood samples

Samples of blood were collected from the retro-orbital venous plexus once every 6 days, two hours after administration of therapeutic agents in the morning. Serum was centrifuged at 16000 rpm for 15 min and analyzed immediately. Levels of aspartate aminotransferase (AST), alanine aminotransferase (ALT), triglyceride (TG), total cholesterol (TC), low-density lipoprotein cholesterol (LDL-C), and high-density lipoprotein cholesterol (HDL-C) in serum were determined by enzymatic colorimetric method. Concentrations of FFA were tested using assay kits purchased from Nanjing Jiancheng Bioengineering Co., Ltd.

### Assessment of LPS levels in the hepatic portal vein

At the end of the treatment, rats were sacrificed using an intraperitoneal injection of 7% chloral hydrate (0.3 mL/100 g). Hepatic portal vein blood samples were centrifuged at 4000 rpm for 10 min at 4°C after collected under anesthetic condition. The concentrations of LPS were measured using a tachypleus amebocyte lysate test purchased from Chinese Horseshoe Crab Reagent Manufactory, Co., Ltd., Xiamen.

### Assessment of TC, TG, lipoprotein, protein, and cytokine levels in TLR4/NF-κB pathway in liver tissue

At the end of the treatment, rats were sacrificed using an intraperitoneal injection of 7% chloral hydrate (0.3 mL/100 g). Tissue samples from the liver were excised and weighed after washing with 0.9% saline. 100 mg tissues were rinsed with PBS and homogenized in 1 mL of PBS and then stored overnight at -20°C. Two freeze-thaw cycles were performed to break all cell membranes, and the homogenates were then centrifuged for 10 minutes at 4000 rpm, 4°C. The supernatant was collected for biochemical analysis. AST, ALT, TG, TC, FFA, LDL-C, and HDL-C concentrations were determined in all liver homogenate samples at the end of the study. Fatty acid transport protein 4 (FATP4), liver-type fatty acid-binding protein (L-FABP), very low-density lipoprotein (VLDL), fetuin-A (FetA), TLR4, NF-κB, tumor necrosis factor-alpha (TNF-α), interleukin-10 (IL-10), interleukin-1 alpha (IL-1α), and interleukin-6 (IL-6) concentrations were tested using ELISA assay kits purchased from Cusabio Biotech Co., Ltd., China.

### Genome-wide expression profiling

Total RNAs from 3 liver tissues (1 from the control group, 1 from the model group, and 1 from the TSG.M group) were harvested using TRIzol (Invitrogen). The RNA samples were amplified and labeled using an Agilent Whole Rat Genome Oligo Microarray (4 × 44 K, Agilent Technologies, Palo Alto, CA, U.S.) using Agilent SureHyb hybridization chambers. After hybridization and washing, the processed slides were scanned with an Agilent microarray scanner G2505C.

The resulting text files were imported into Agilent GeneSpring GX software (version 11.0) for further analysis. The microarray data sets were normalized in GeneSpring GX using the Agilent FE one-color scenario. Differentially expressed genes were identified by determining the fold-change (FC). Functional analysis of differentially expressed genes was performed using gene ontology (GO) (http://www.geneontology.gov/) [26] and the KEGG PATHWAY Database (http://www.genome.jp/kegg/pathway.html).

### Measurement of SIBO

Here, 45 mg sample of intestinal content in each rat was weighed. All the intestinal content samples in the same group were carefully blended and 45 mg of mixed sample was weighed and diluted in 30 mL of sterile saline and incubated at 37°C at 180 rpm for 0.5 h. Then 50 μL of the supernatant was coated on blood agar plates and then incubated at 37°C for 72 h. Total bacterial count over 10^5^ colony forming units (CFU) per mL here served as a diagnostic criterion of small intestinal bacterial overgrowth (SIBO).

### Flow cytometric analysis of occludin and ZO-1 protein levels

Single-cell suspension was prepared by cutting and disrupting one representative intestine sample in each group through 70 μm filter membrane. Cells were diluted with staining buffer to 1 × 10^7^ cells/mL after blockage with 3% FBS. For analysis, cells were incubated with anti-occludin antibody (Abcam, U.S.) and anti-ZO-1 antibody (Proteintech, U.S.) for 2 h and then incubated with fluorescein isothiocyanate (FITC) in the dark for 1 h. And then the expressions of occludin and ZO-1 were tested using flow cytometry (FACSCalibur, Becton, Dickinson, and Company, U.S.).

### Overall structural changes in gut microbiota

One fecal sample from each group in the 1^st^ and 12^nd^ week was chosen for pyrosequencing of V4 regions of 16S rDNA, respectively. The protocol described by Caporaso et al. was here used to assess the diversity and composition of the bacterial communities in each fecal sample [27]. PCR amplification was conducted with the 515f/806r primer set, and the V4 region of 16S rDNA gene was amplified. The reverse primer contained a 6 bp error-correcting barcode unique to each sample. DNA was amplified using a previously described protocol [28]. Sequencing was conducted on an Illumina MiSeq platform.

### Statistical analysis

The data (mean ± SD) were evaluated by one-way analysis of variance (ANOVA) with a significance level of *P* < 0.05, < 0.01, and < 0.001.

## Results

### TSG and the general physiological features

**Fig 1:**
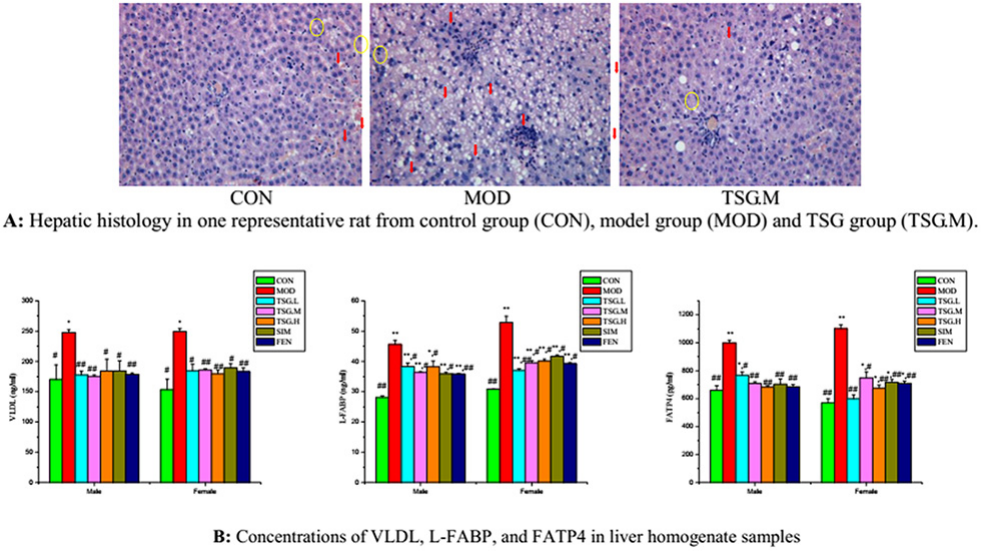
Hepatic histology and expressions of VLDL, L-FABP and FATP4 in liver tissues. (**A**) **Representative images (200×magnification, haematoxylin and eosin stain) of hepatic histology in CON, MOD and TSG.M groups.** No obvious fatty degeneration was observed in hepatocytes of CON group. Obvious edema (yellow cycles) and steatosis (red arrows) were observed in hepatocytes after high fat diet fed for12weeks.These edema and steatosis were markedly relieved in TSG.M group. **(B) Concentrations of VLDL, L-FABP, and FATP4 in liver homogenate samples.** The bars represented protein levels of VLDL, L-FABP, and FATP4 in liver homogenate samples, which were determined by ELISA in all groups (mean ± SD, n = 7). Higher experssions of VLDL, L-FABP, and FATP4 were found in MOD group due to the high fat feed induction.TSG treatment could significantly reduce their expressions. Statistical significance: * p< 0.05 vs. control;** p< 0.01 vs. control; ^#^ p< 0.05 vs. model; ^##^ p< 0.01 vs. mode.

The initial body weights of the male and female rats were similar (215 ± 5 g), and the daily food intake was similar among all groups. There was no difference in the body weight growth rate between normal food feed groups and HFD groups. However, weight growth showed distinct gender differences. TSG treatments did not produce a sharp decline in appetite and weight in rats. The liver index in the HFD group increased significantly (*P* < 0.01) (Table 1) with obvious edema and steatosis (Fig 1A).

**Table 1:** Body weight, index of liver and some lipid indexes in the liver samples. (mean ± SD, n = 7)

	CON	MOD	TSG.L	TSG.M	TSG.H	SIM	FEN
♂Body weight (g)	404.2±56.95	454.7±43.41	474.4±65.00	457.7±50.74	464.0±42.27	433.3±68.45	428.3±32.22
♂Index of liver (%)	2.22±0.17^###^	3.48±0.15***	3.00±0.36**	2.78±0.29** ^,^ ^##^	2.70±0.43* ^,^ ^#^	3.37±0.69**	3.59±0.32***
♂TC level (mmol/L)	0.71±0.01^###^	1.29±0.00***	1.00±0.01* ^,^ ^###^	0.86±0.01* ^,^ ^###^	0.80±0.01* ^,^ ^###^	0.78±0.01* ^,^ ^###^	0.86±0.00* ^,^ ^###^
♂TG level (mmol/L)	1.06±0.04^##^	1.73±0.00**	1.28±0.08^#^	1.45±0.01* ^,^ ^###^	1.36±0.04* ^,^ ^#^	1.09±0.00^###^	1.49±0.01* ^,^ ^###^
♂FFA level (μmol/L)	126.9±4.257^##^	242.9±9.122**	171.1±6.081* ^,^ ^#^	122.6±18.85^#^	124.3±1.824^##^	134.2±12.16^##^	168.1±12.77* ^.^ ^#^
♂LDL-C level (mmol/L)	1.36±0.11**	2.64±0.08^##^	1.59±0.06^##^	1.64±0.11^##^	1.45±0.06^##^	1.71±0.11^#^	1.82±0.06* ^,^ ^#^
♂HDL-C level (mmol/L)	1.27±0.06^#^	0.89±0.09*	1.10±0.10	1.10±0.35	1.12±0.04	1.01±0.04*	1.08±0.09
♂AST level (U/L)	2728±125.6^##^	4185±100.3**	3213±121.1^#^	3156±195.6^#^	3251±110.8* ^,^ ^#^	3697±134.6*	3549±77.61* ^,^ ^#^
♂ALT level (U/L)	1232±119.3^#^	1921±100.4*	1451±128.5	1361±100.5^#^	1468±124.7	1669±145.7	1601±116.6
♀Body weight (g)	294.0±19.42	282.4±17.24	307.2±36.99	287.4±30.58	291.0±27.28	301.4±23.92	316.1±20.35^#^
♀Index of liver (%)	2.44±0.30^##^	3.36±0.46**	2.74±0.28^#^	2.70±0.34^#^	2.99±0.40*	2.90±0.41*	3.32±0.38***
♀TC level (mmol/L)	0.55±0.01^###^	1.05±0.08***	0.71±0.00* ^,^ ^###^	0.77±0.02* ^,^ ^#^	0.84±0.01* ^,^ ^#^	0.93±0.01*** ^,^ ^#^	0.83±0.01* ^,^ ^#^
♀TG level (mmol/L)	1.06±0.02^##^	1.49±0.01**	1.14±0.01* ^,^ ^###^	1.24±0.01** ^,^ ^##^	1.20±0.01* ^,^ ^###^	1.25±0.03* ^,^ ^##^	1.20±0.08^#^
♀FFA level (μmol/L)	52.89±15.20^#^	153.1±10.95*	73.10±20.68^#^	108.4±4.865* ^,^ ^#^	86.43±15.20^#^	78.69±16.42^#^	84.71±11.55^#^
♀LDL-C level (mmol/L)	1.42±0.09^##^	2.53±0.04**	1.72±0.10^##^	1.75±0.09^##^	1.77±0.04* ^,^ ^##^	1.77±0.06* ^,^ ^##^	1.69±0.08^##^
♀HDL-C level (mmol/L)	1.40±0.08^#^	0.86±0.05*	1.29±0.03^##^	1.23±0.06^#^	1.18±0.08^#^	1.19±0.01^##^	1.21±0.03^#^
♀AST level (U/L)	3183±104.6^##^	5352±122.9**	4162±74.37** ^,^ ^##^	4352±93.15** ^,^ ^#^	4342±187.5* ^,^ ^#^	4375±140.1* ^,^ ^#^	4528±62.88** ^,^ ^#^
♀ALT level (U/L)	1437±120.3^#^	2465±107.7*	1678±129.2^#^	1664±142.0^#^	1660±184.3^#^	1775±163.8	1793±61.72

CON: normal control; MOD: HFD model; TSG.L: low dosage of TSG; TSG.M: middle dosage of TSG; TSG.H: high dosage of TSG; SIM: simvastatin; FEN fenofibrate. The * indicates a significant difference compared with control group. The ^#^ indicates a significant difference compared with model group.

* p < 0.05

** p < 0.01

*** p < 0.001.

^#^ p < 0.05

^##^ p < 0.01

^###^ p < 0.001.

Levels of total cholesterol (TC), triglyceride (TG), FFA, low-density lipoprotein cholesterol (LDL-C), high-density lipoprotein cholesterol (HDL-C), aspartate aminotransferase (AST), alanine aminotransferase (ALT), very-low-density lipoprotein (VLDL), liver type fatty acid binding protein (L-FABP), and fatty acid transport protein 4 (FATP4) in liver tissue were tested after execution at the end of the study (Table 1 and Fig 1B). HFD increased the TC and TG levels by 81.7% (89.8%) and 63.3% (40.5%) separately in liver tissue of the male group and female group, with female group results shown in parentheses. These results indicated that the NAFLD model had been successfully established in the current study. TSG was found to reduce the concentrations of FFA, TG, and TC in the liver and control the extent of liver hypertrophy effectively (Fig 1A). The elevations of AST and ALT induced by HFD were also alleviated by TSG, and they were even slighter than those of simvastatin and fenofibrate. In male groups, the FFA level in the high dose (TSG.H) group was significantly lower, possibly mediated by the decrease in L-FABP and FATP4 expression. These are the predominant transportation and binding proteins of FFA. TSG was found to inhibit the accumulation of FFA in liver, which cuts off the supply of raw TG materials for endogenous synthesis. However, TSG reduced the accumulation of TG in the liver by lowering the expression of VLDL and LDL-C and increasing the concentration of HDL-C. The alleviation of NAFLD by TSG was even more pronounced in female rats.

### TSG and regulation of intestinal microbial balance and overgrowth

The relationships between the gut microbiota imbalance and NAFLD pathogenesis are widely recognized [29]. HFD induced specific variations in gut microbiota, which contain many *Firmicutes* but fewer *Bacteroidetes*. The number of individuals from phyla *Firmicutes* and *Bacteroidetes*, which represent more than 90% of the total gut microbiota, were higher in NAFLD individuals.

**Fig 2:**
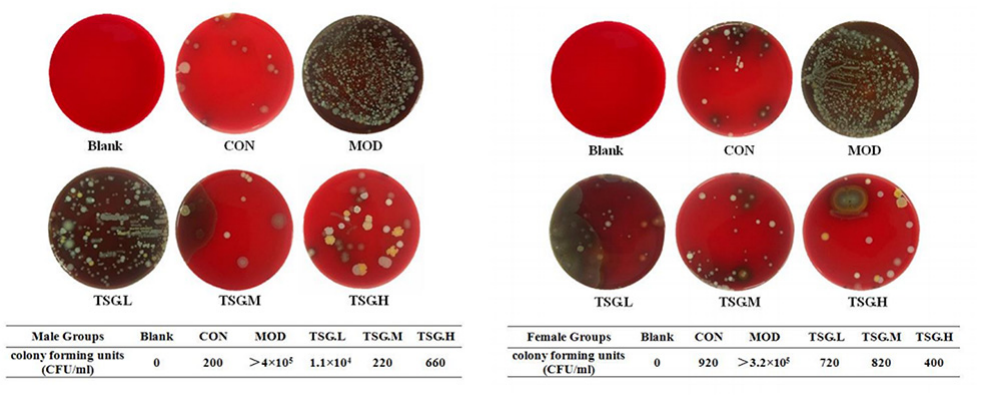
SIBO inhibition effects of TSG. Blended intestinal content sample in each group (45 mg) was cultivated at 37°C for 72 h on blood agar plates. The numbers of colonies were significantly higher in MOD group than CON group in both sexes. TSG treatment reduced the numbers of colonies in a dose-dependent manner. In the meantime, hemolysis extent was also alleviated after TSG treatment. Colony counts (CFU/mL) of each sample were listed below.

At the end of the study, the intestinal contents of rats were cultivated for 72 h on blood agar plates (Fig 2). The number of colonies was significantly higher in the model group, whose members also experienced obvious hemolysis. SIBO was diagnosed in the model group and found to be inhibited in the TSG groups in a dose-dependent manner.

**Fig 3:**
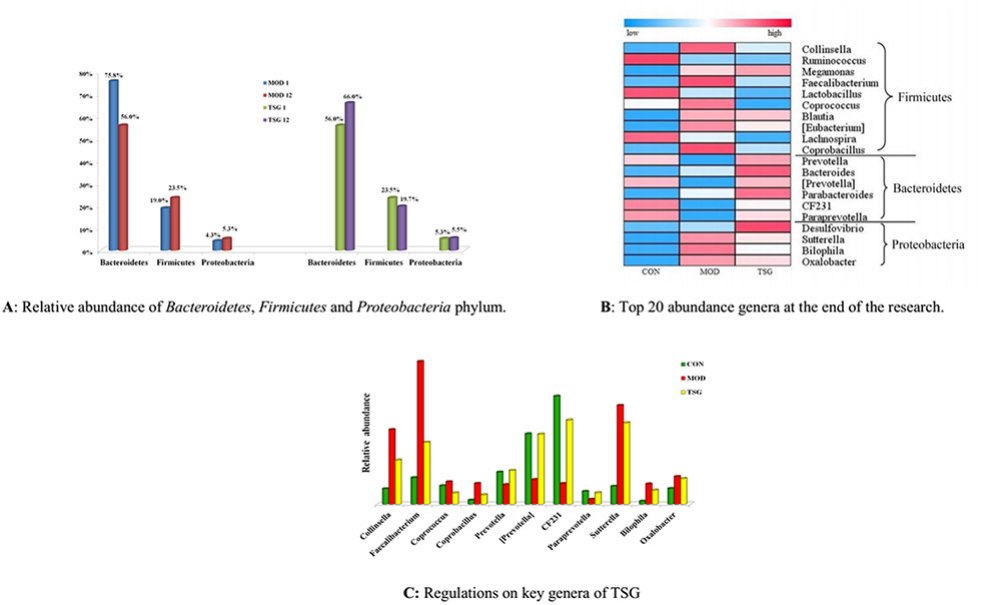
TSG and intestinal microbial balance regulation. **(A) Relative abundance of *Bacteroidetes*, *Firmicutes* and *Proteobacteria* phylum.** One fecal sample from each group in the 1^st^ and 12^nd^ week was chosen for pyrosequencing of V4 regions of 16S rDNA, respectively. Blue bars and red bars represented the relative phylum abundance of male MOD group in the 1^st^ and 12^nd^ week, respectively. Green bars and purple bars represented the relative phylum abundance of male TSG.M group in the 1^st^ and 12^nd^ week, respectively. (**B**) **Top 20 abundance genera at the end of the research**. Heat map was showing the abundance of top 20 abundance genera in male CON, MOD and TSG.M group. Genera in *Bacteroidetes and Proteobacteria* phylum had relative higher abundance, while genera in *Firmicutes* had relative lower abundance after TSG treatment. **(C) Regulations on key genera of TSG.** Green, red and yellow bars displayed specific genera abundance in male CON, MOD and TSG.M group at the end of the research.

In this study, HFD was found to increase the relative abundance of *Firmicutes* and *Proteobacteria* by 4.60% and 1.03%, respectively, and to reduce the abundance of *Bacteroidetes* from 75.8% to 56.0% in the male group (Fig 3A). These results indicated that the changes in the intestinal microbial balance may be an environmental factor of obesity and NAFLD [22]. These alterations were reversed by TSG treatment (Fig 3A). Relative abundance of top 20 genera (Fig 3B) showed that the TSG could increase *Prevotella*, *[Prevotella]*, *CF231*, and *Paraprevotella* to normal levels effectively. These genera belong to the *Bacteroidetes* phylum. TSG, meanwhile, also reduced the relative abundance of some genera of *Firmicutes* phylum, such as *Collinsella*, *Faecalibacterium*, and *Coprobacillus* (Fig 3C).

There were far more microbes from phylum *Proteobacteria*, especially genus *Bilophila*, among the microbiota from the NAFLD group than in the healthy group [23, 29]. TSG was found to decrease the relative abundance of *Bilophila* and other genera in phylum *Proteobacteria* effectively.

### TSG and enterogenous endotoxin and intestinal mucosal barrier regulation

**Fig 4:**
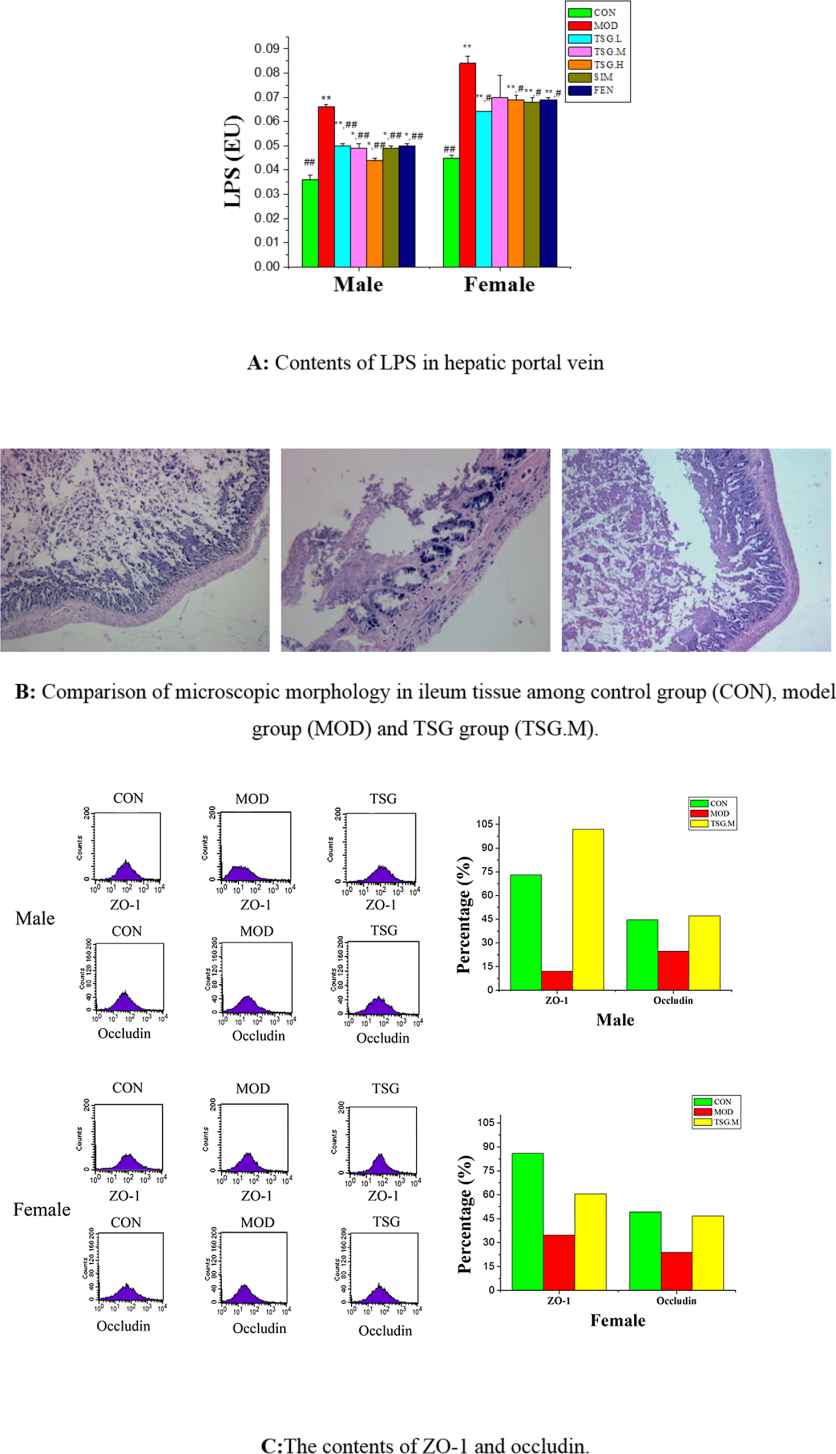
TSG and enterogenous endotoxin and intestinal mucosal barrier. **(A) Contents of LPS in hepatic portal vein.** LPS levels in hepatic portal vein blood samples were measured using a tachypleus amebocyte lysate test (mean±SD, n = 7). Statistical significance: * p< 0.05 vs. control;** p< 0.01 vs. control;*** p< 0.001 vs. control; ^#^ p< 0.05 vs. model; ^##^ p< 0.01 vs. model; ^###^ p< 0.001 vs. model. **(B) Comparison of microscopic morphology in ileum tissue among CON, MOD and TSG.M group**. Secional representations (100 ×magnification, haematoxylin and eosin stain) of ileum tissues. Degeneration and necrosis of ileum were relieved after TSG treatment. **(C) The contents of ZO-1 and occludin.** Expressions of occludin and ZO-1 in one representative intestine sample in each group were tested using flow cytometry, respectively. Single-cell suspensions were incubated with anti-occludin and anti-ZO-1 antibody for 2 h and then incubated with FITC in the dark for 1 h.Tight junctions proteins, such as occludin and ZO-1, were dramatically reduced in high fatd diet fed rats. These reductions might be lighted

LPS, a cell-wall component of gram-negative bacteria, delivered to the liver via the portal vein in endotoxemia. Such endotoxin production by gut microbiota can cause chronic low-grade inflammation in patients with NAFLD. After the rats were fed HFD for 12 weeks, the LPS level in NAFLD group was significantly higher than in the normal group (*P* < 0.01) in both sexes (Fig 4A). Fortunately, TSG showed a satisfactory effect with respect to the inhibition of the rise of LPS, in a dose-dependent manner, especially among male rats. This inhibition effect may be related to its beneficial effects on gut microbiota equilibrium.

Intestinal epithelial cells establish a barrier between hostile external environments and the internal milieu. The integrity of small intestinal mucosa is frequently disrupted in a variety of acute or chronic intestinal diseases [30]. Tight junction proteins of small intestinal mucosa such as zonula occludens (ZO) and occludin are critical to the maintenance of the intestinal epithelial barrier [31].

In the current study, degeneration and necrosis were visible in the intestinal mucosa epithelial tissue in NAFLD rats (Fig 4B). Treatment of TSG was found to minimize the damage and maintained the structural integrity.

The protein expressions of ZO-1 and occludin were significantly lower in the model group than in the normal group (Fig 4C), which was in agreement with the findings of a previous study [31]. At the same time, TSG raised ZO-1 and occludin protein expression to normal levels. In general, TSG was found to improve the function of intestinal mucosal barrier, increase the expression of the ZO-1 and occludin protein, and inhibit endotoxin-translocation-induced endotoxemia.

### TSG and suppression of the TLR4/NF-κB pathway

**Fig 5:**
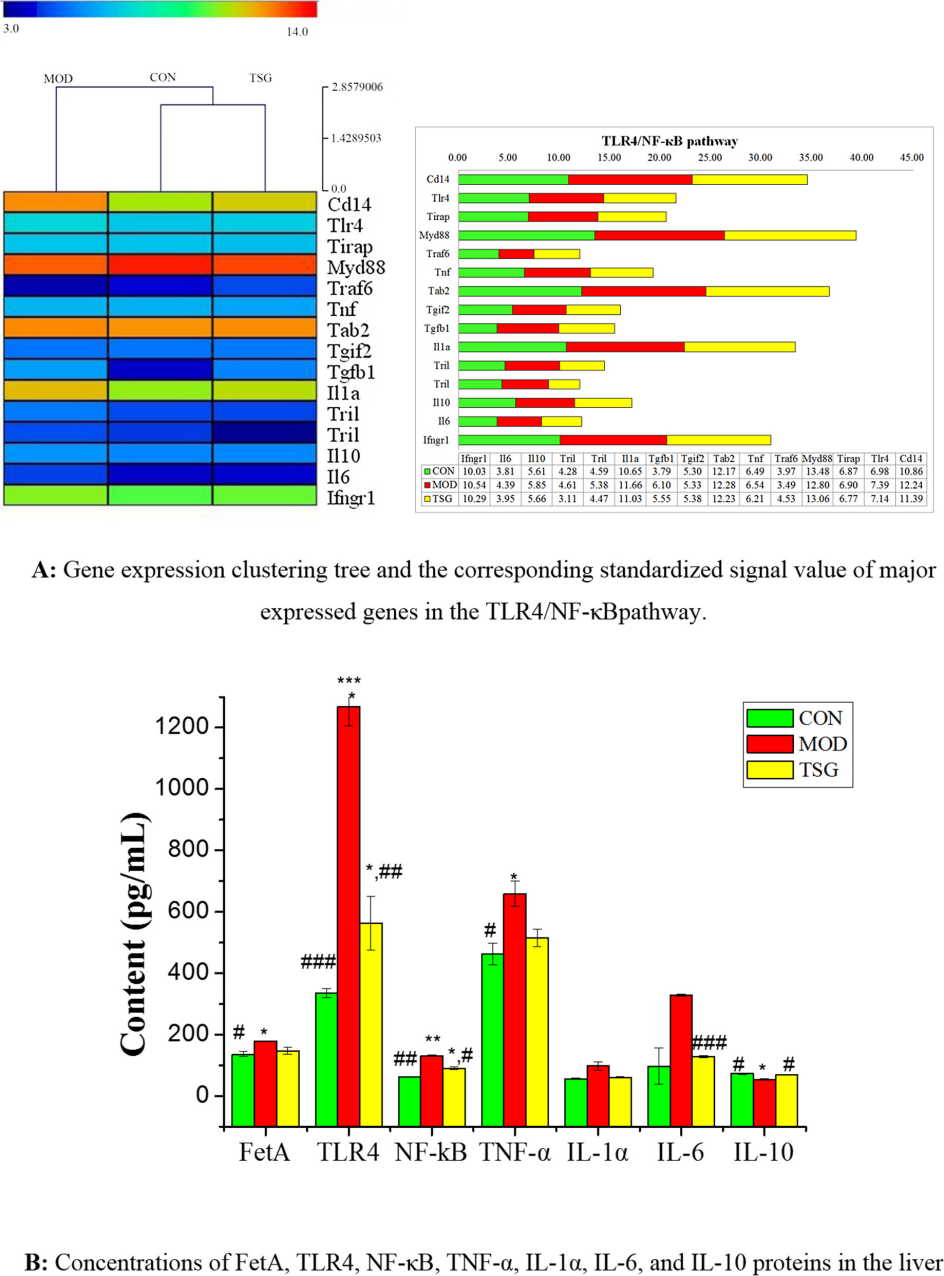
Rat liver tissue gene and protein expression in TLR4/NF-κB pathway. **(A) Gene expression clustering tree and the corresponding standardized signal value of major expressed genes in the TLR4/NF-κB pathway**. Total RNA from 3 liver tissues (1 from the control group, 1 from the model group, and 1 from the TSG.M group) were harvested, amplified and labeled using an Agilent Whole Rat Genome Oligo Microarray (4 × 44 K) using Agilent SureHyb hybridization chambers. The processed slides were scanned with an Agilent microarray scanner G2505C after.hybridization and washing. Agilent Feature Extraction software was used to analyze acquired array images. Quantile normalization and subsequent data processing were performed with using the GeneSpring GX v11.5.1 software package. After quantile normalization of the raw data, genes that at least 1 out of 3 samples have flags in Detected (“All Targets Value”) were chosen for further data analysis. Differentially expressed genes between the two samples were identified through Fold Change filtering. Hierarchical Clustering was performed using the R scripts. **(B) Concentrations of FetA, TLR4, NF-κB, TNF-α, IL-1α, IL-6, and IL-10 proteins in the liver.** Tissue samples from the liver were excised and weighed after washing with 0.9% saline after rats were sacrificed using an intraperitoneal injection of 7% chloral hydrate (0.3 mL/100 g).100 mg tissues were rinsed with PBS and homogenized in 1 mL of PBS and then stored overnight at -20°C. Two freeze-thaw cycles were performed to break all cell membranes, and the homogenates were then centrifuged for 10 minutes at 4000 rpm, 4°C. The supernatant was collected for analysis. Concentrations of FetA, TLR4, NF-κB, TNF-α, IL-1α, IL-6, and IL-10 were tested using ELISA assay kits (mean±SD, n = 7). Statistical significance: * p< 0.05 vs. control;** p< 0.01 vs. control;*** p< 0.001 vs. control; ^#^ p< 0.05 vs. model; ^##^ p< 0.01 vs. model; ^###^ p< 0.001 vs. model

Rat liver tissue gene spectrum scanning of three groups was performed using 4 × 44 k gene expression in rats with Agilent production chips. The gene expression clustering tree and the corresponding standardized signal value of major expressed genes in TLR4 inherent immune response system are shown in Fig 5A.

HFD promoted the CD14 and TLR4 signaling expression and the combination of the ligand and CD14/TLR4. The expressions of some downstream signaling molecules, such as TNF, beta activated protein kinase binding protein Tab2, transforming growth factor beta (Tgfb1), IL-1α, IL-6, and interferon gamma receptor 1 (Ifngr1) signal molecules, were increased, and this triggered the TLR4 inherent immune response system. The expression of these genes was downregulated by TSG.M treatment.

TSG down-regulated TLR4 gene expressions and reduced 56% TLR4 protein expression relative to the NAFLD model group. Chronic low-grade inflammation, characterized by the over-release of inflammatory cytokines such as TNF-α, IL-1α, IL-6, IL-10, were also controlled by TSG (Fig 5B). All these findings indicated that TSG decreased the activation of the TLR4/NF-κB inherent immune response system, which could delay and restrict the occurrence and development of NAFLD.

## Discussion

Regarding the anatomy of the liver and intestinal homology, as the close intercommunication of the metabolism and the immune system has become better understood, the concept of the “gut-liver axis” has gradually been put forward and accepted. This concept addresses the close relationships between the liver, gut, cells, cytokines, and other factors in the portal vein system and how they influence each other. Several studies indicate that patients with liver disease often have visible intestinal mucosal barriers and the destruction of the intestinal mucosal barrier function is directly or indirectly related to various types of liver damage and diseases of the whole body. Improving the intestinal mucosal barrier is helpful to the treatment of liver disease.

Modern studies have shown that disorders of the intestinal microenvironment disorders play a key role in the pathogenesis of liver disease and in changes in the relative proportion and intestinal micro ecology, such as the excessive breeding of intestinal flora (SIBO), which has a key role in NAFLD. The theory of the gut-liver axis can partially explain the pathogenesis of NAFLD, the disorder of the intestinal flora, and damage to the intestinal mucosa barrier, which triggers the innate immune response in the liver, which disrupts the liver’s steady-state and aggravates liver inflammation. PAMPs can activate the toll-like receptor 4 (TLR4), signaling mediated by TLRs, and the release of cytokines, such as MyD88, interferon (IFN), and then promote the combination of LPS and CD14/TLR4. In the liver, LPS/TLR4 leads to the unusual release of large amounts of cytokines such as transforming growth factor-β (TGF-β) by liver cells. Therefore, TGF-β is directly related to liver fibrosis.

In this work, the activity and mechanisms of TSG on the prevention and treatment of NAFLD were investigated. The results are as follows: First, TSG was found to affect the intake of lipids from food and reduce the accumulation of FFA effectively and to do so primarily by reducing the expression of L-FABP and FATP4. L-FABP, a small cytosolic protein, is expressed in various tissues such as the liver, small intestines, and kidneys. L-FABP is responsible for the transport and metabolism of intracellular fatty acids [32]. FATP4 is the only FATP expressed in the apical brush border of the small intestine, where it is thought to be responsible for absorption of dietary lipids [33]. HFD can induce and promote the expression of L-FABP and FATP4. The expression of these genes is significantly altered without visible pathological changes to liver tissues [34]. A previous study has shown that TSG reduces the *in vitro* expression of both L-FABP and FATP4 [35]. The *in vivo* results of this study have confirmed these benefits.

Secondly, TSG reduced LPS of the portal vein by nearly 25%. The regulation of gut microbiota balance and strong intestinal mucosal barrier mediated by TSG may be responsible for this. SIBO with obvious hemolytic phenomena were also inhibited by the treatment of TSG. Intestinal mucosa integrity was found to prevent LPS-induced endotoxemia. The increasing expression of ZO-1 and occlusion, which was observed in epidermal tight junctions, rebuilt the tissue structure of intestinal mucosal epithelial cells [36]. This protective effect on intestinal mucosa was confirmed by light microscopic observation.

Subsequently, the gene and protein expression of TLR4 and NF-κB were inhibited by TSG. TSG was found to reduce expression of TLR4 by 56% and that of NF-κB by 23% relative to the HFD model group. The release of downstream inflammatory cytokines in the LPS/TLR4/NF-κB signaling pathway was mitigated by TSG. TLR4 signaling plays pivotal roles in the pathogenesis of non-alcoholic steatohepatitis [37]. TLRs and its downstream molecules mediate steatosis, inflammation, and fibrosis [7]. Activation of the transcriptional factor NF-κB, a downstream target of TLR-MyD88 signaling, is crucial to the inflammatory response in immune cells and is key to the development of NAFLD [11, 38]. Increased expression of various pro-inflammatory mediators such as TNFα, IL-1α/β, IL-6, and others is also crucial to the development of NAFLD [39]. In this way, the regulation of TLRs and their downstream molecules by TSG indicates that it may be a suitable therapy for NAFLD.

In summary, these findings demonstrate that the natural stilbenoid TSG can reverse the occurrence and development of NAFLD with multi-conditioning in the gut-liver axis. These results suggest that TSG may serve as a promising lead compound or an intervention in NAFLD therapy.

We found some tiny difference in the results between male and female rats, however, we could not say the effects of TSG are gender-based or there is any gender difference, currently. Further researches about the effects of TSG on NAFLD rats are still needed for more in-depth interpretation.
